# Pause and utterance duration in child-directed speech in relation to child vocabulary size*

**DOI:** 10.1017/S0305000914000609

**Published:** 2014-10-21

**Authors:** ULRIKA MARKLUND, ELLEN MARKLUND, FRANCISCO LACERDA, IRIS-CORINNA SCHWARZ

**Affiliations:** Department of Linguistics, Stockholm University

## Abstract

This study compares parental pause and utterance duration in conversations with Swedish speaking children at age 1;6 who have either a large, typical, or small expressive vocabulary, as measured by the Swedish version of the McArthur-Bates CDI. The adjustments that parents do when they speak to children are similar across all three vocabulary groups; they use longer utterances than when speaking to adults, and respond faster to children than they do to other adults. However, overall pause duration varies with the vocabulary size of the children, and as a result durational aspects of the language environment to which the children are exposed differ between groups. Parents of children in the large vocabulary size group respond faster to child utterances than do parents of children in the typical vocabulary size group, who in turn respond faster to child utterances than do parents of children in the small vocabulary size group.

## INTRODUCTION

It is widely accepted both that linguistic input during infancy and childhood influences early language development, and that parents modify their speech when talking to children. Most recently, it has been shown that it is primarily the amount of child-directed speech that has a positive effect on children's language development, not adult-directed speech overheard by the child (Weisleder & Fernald, 2013). In the light of these findings, it is more important than ever to study the types of modification that parents make when speaking to children, and the relationship between these behaviours and children's language development.

A substantial relationship between different aspects of parental input and child language skill has been demonstrated in several earlier studies. Whereas some have focused on the content of the linguistic input, many have demonstrated that even simple quantitative measures are of importance in this line of study. For example, the number of utterances in mothers' speech to their children at age 1;6 has been shown to be related to child receptive vocabulary size at two years of age (Hurtado, Marchman & Fernald, 2008). Although the authors also analyzed qualitative content by measuring the number of word tokens and word types, as well as mean length of utterance, it was the simple quantitative measure of number of utterances that was most strongly correlated with child vocabulary size (see also Hoff, 2003; Hoff & Naigles, 2002). Mothers have also been shown to differ in the amount of speech and number of questions directed to their children at 1;11 to 2;1, depending on the children's productive vocabulary size (Smolak & Weintraub, 1983). Mothers of children with fewer than sixty words in their vocabularies used smaller amounts of speech (as measured in total number of utterances and words produced) than did mothers of children with vocabularies of at least 100 words. In this case, child vocabulary size was estimated on the basis of interviews with the mothers. Additionally, the number of mothers' individual word tokens has been shown to be related to vocabulary growth in children between 1;2 and 2;2. Children with large vocabularies and rapid vocabulary growth, estimated on the basis of longitudinal observational sessions, had mothers who used a greater number of word tokens than children with smaller vocabularies and a slower vocabulary growth rate (Huttenlocher, Haight, Bryk, Seltzer & Lyons, 1991).

Temporal contingency of mothers' responses, both linguistic and non-linguistic in nature, has also been shown to influence early language development. In a study by Goldstein, King, and West (2003), mothers were asked to smile at, move closer to, and touch their eight-month-old infant at specific times, either directly after an infant vocalization or at time-points unrelated to the infant's vocal behaviour. Vocal productions of the infants were then rated for maturity (in terms of, e.g. precanonical vs. canonical babbling), and infants in the contingent conditions increased the number of canonical syllables whereas infants in the non-contingent group did not. A similar procedure also incorporating different types of vocal behaviour of the parent have been shown to influence the phonology of infant vocalizations in the contingent condition but not in the non-contingent condition (Goldstein & Schwade, 2008), indicating that the temporal alignment of responses to child vocalizations influences early language development.

The growing body of research on infant- and child-directed speech (IDS and CDS) clearly shows that parents modify their speech when talking to children. In fact, all adults measurably adjust the way they speak when talking to a child (e.g. Ferguson, 1964; Soderstrom, 2007). This adjustment is for example characterized by a reduction of linguistic complexity (Papoušek, Papoušek & Haekel, 1987) and prosodic (Fernald & Simon, 1984; Grieser & Kuhl, 1988) and phonetic (Kuhl *et al.*, 1997; Sundberg & Lacerda, 1999) modifications. In addition, temporal-durational characteristics of speech are modified in CDI, such as the duration of utterances and pauses. In CDS, utterance duration is typically shorter compared to that of adult-directed speech (ADS) (e.g. Jaffe, Beebe, Feldstein, Crown & Jasnow, 2001). This has been reported for several languages, including Mandarin (Grieser & Kuhl, 1988), Dutch (van de Weijer, 1997), German (Fernald & Simon, 1984), and American English (Beebe, Alson, Jaffe, Feldstein & Crown, 1988). Parental utterance duration varies with the age of the child: the older the child, the longer the utterance (Stern, Spieker, Barnett & MacKain, 1983). Pause duration is typically longer in CDS than in ADS (Fernald & Simon, 1984). This pattern is found across languages (Fernald, Taeschner, Dunn, Papousek, de Boysson-Bardies & Fukui, 1989) and varies with child age; the longest pauses are found during the neonatal period, becoming shorter with increasing child age (Stern *et al.*, 1983).

One central aspect of vocal interaction is turn-taking. Speaker and listener in a conversation take turns, often making switching pauses at turn exchanges. A turn exchange consists of three elements: an utterance from the first speaker, a switching pause (or a short period of overlapping speech), and an utterance from the second speaker. Typical conversations consist of a number of turns, with the participants alternating as speaker and listener (e.g. Duncan, 1972; Jaffe & Feldstein, 1970; Sacks, Schegloff & Jefferson, 1974). Participants in typical adult–adult interaction have been shown to match both intrapersonal and switching pause duration to that of their conversational partner during the course of the conversation (e.g. Edlund, Heldner & Hirschberg, 2009; Jaffe & Feldstein, 1970). Vocal turn-taking behaviour has also been described in interactions between parents and infants (Bateson, 1975; Jasnow & Feldstein, 1986; Velandia, Matthisen, Uvnäs-Moberg & Nissen, 2010) as well as between parents and children (Welkowitz, Bond, Feldman & Tota, 1990). As mentioned above, temporal aspects of turn-taking such as utterance and pause duration are spontaneously modified when adults speak to infants and children. Some studies differentiate between switching and intrapersonal pauses: switching pauses are pauses at turn exchanges, while intrapersonal pauses take place within one turn; that is the same person speaks before and after the pause. However, both pause types are longer in CDS than in ADS (Beebe *et al.*, 1988). Mutual matching of switching pauses has also been found in parent–infant interaction (Beebe *et al.*, 1988; Jasnow & Feldstein, 1986), as well as in parent–child interaction with four- and five-year-olds (Welkowitz *et al.*, 1990).

To summarize, durational modifications that adults make in their speech when talking to a child can affect the child's language development. To increase the body of knowledge on durational modifications and their relationship to child vocabulary size, the present study investigates if and how Swedish parents modify utterance and pause duration in vocal interaction with their children at 1;6, comparing the durational behaviour of parents to children with large, typical, and small vocabularies. In contrast to the studies cited above, in which parental input is synonymous with maternal speech, the present study does not differentiate between maternal and paternal input. Instead, the input of both mothers and fathers is combined as parental speech. It is typical for Swedish fathers to take parental leave and stay at home with their children, as the government encourages parents to share the allotted 18 months equally. The present study focuses on the quantitative, and therefore objectively measureable speech aspects of utterance and pause duration. Since parental utterance and pause modification is of interest, the analysis is based exclusively on instances when the second utterance in an utterance–pause–utterance sequence is produced by a parent. When the speaker of the first utterance is a child, and the pause therefore is a child–parent switching pause, the utterance–pause–utterance sequence is henceforth called a child–parent turn-taking event. When the speaker of the first utterance is a parent, the pause is either an intrapersonal pause within a parent-produced monologue, or a parent–parent switching pause. Henceforth, these sequences are called parent–parent intrapersonal turn-taking event or parent–parent switching turn-taking event. Parental second utterance duration and pause duration in child–parent turn-taking events are expected to differ according to child vocabulary size.

## METHOD

### Material

The speech material analyzed in the present study comprised audio-recordings of spontaneous parent–child interaction, collected within the SPRINT project, a prospective longitudinal language development study in which families took part in an intervention programme to support children's communicative development. Sixty baseline recordings were included, featuring fifteen children: eight girls and seven boys. At the time of the recordings, the children were aged between 1;5·8 and 1;6·2. Parents recorded spontaneous interaction in four different types of everyday situation in their family home: mealtime, playtime, reading time, and getting dressed. They used a digital audio recorder (ZOOM Handy Recorder H2). At least one parent and the child were present in each recorded interaction, but at times both parents and/or one or two siblings were also present (see Table 1 for details). For each child, all recordings were made within a period of eight days. Typically, recording sessions lasted for 20 to 30 minutes, and parents uploaded the recordings to the project database.

**Table 1.** Number of audio-recordings per speaker constellation: speaker constellation in the audio-recordings varied across vocabulary size groups since the recordings contain authentic daily life interactions. The table shows the number of recordings per speaker constellation and vocabulary size group.

Vocabulary size	Child and one parent	Child and both parents	Child, one parent, and one or two siblings	Child, both parents, and one sibling
**Large**	12	7	–	1
**Typical**	13	4	3	–
**Small**	10	7	–	3
**Sum**	35	18	3	4

Selection of recordings for the present study was based on the children's productive vocabulary size at the age of 1;6. Vocabulary size was measured by the Swedish version of the MacArthur-Bates Communicative Development Inventory (Berglund & Eriksson, 2000; Fenson *et al.*, 1993). The inventory ‘Words and sentences’, normed for Swedish children from 1;4 to 2;4, was administered in an on-line version (Marklund, 2009).

The sixty recordings were selected from pre-intervention data of the SPRINT project and featured children with vocabulary scores either in the highest (90–100%), upper-medium (50–75%), or lowest (0–25%) percentile range, five in each category. First, the five children with the lowest vocabulary scores were selected to then find matches among the children with the highest and upper-medium scores. Matching criteria were gender, number and age of siblings, birth order, and the use of Swedish as the native language spoken at home (non-exclusive). All fifteen children included in the study were born full-term with normal birth weight. Among the children with typical vocabulary size, two had suffered from ear infections during their first year of life but with no resulting hearing problems, and one was reported to have an older sibling with language difficulties. Children within the highest percentile range had a large vocabulary size for their age. They were in fact at the level of children at 1;10 to 2;4 with typically sized vocabularies if matched according to vocabulary proficiency (Berglund & Eriksson, 2000). Children in the upper-medium percentile range had typical vocabulary size, and children within the lowest percentile range had small vocabulary sizes for their age. Children with small vocabularies form a potential risk group for a later diagnosis of language impairment.

### Procedure

Five minutes of each recording were selected for analysis, starting at the onset of the first parent utterance classified as child-directed. For each speaker in the recording, onset and offset were manually marked for all utterances considered to have a communicative purpose (e.g. coughing and laughing were excluded). Utterance on- and offsets were sorted by a time stamp within each recording, and pauses were defined as periods of time during which no utterance occurred. On- and offset of utterances surrounding any pause were extracted, as was speaker identity. All utterance and pause durations were collected from the entire sample of sixty recordings to calculate the mean duration of pauses and surrounding utterances. These were defined as units within a turn-taking event. Turn-taking events including a sibling or any adult other than a parent were excluded from the analysis. Since parental durational modifications are the subject of this study, pauses followed by parent utterances were of particular interest, as parent behaviour determines their duration. Consequently, only turn-taking events in which a parent is the speaker of the second utterance were included in further analyses. Therefore, three types of pauses were studied: (i) child–parent switching pauses: the pauses following a child utterance and preceding a parent utterance; (ii) parent–parent intrapersonal pauses: pauses between two utterances from the same parent; and (iii) parent–parent switching pauses: pauses following an utterance from one parent and preceding an utterance from the other parent. The duration of utterances preceding and following these types of pause was studied. For a more in-depth analysis, parental utterances were transcribed. This was done to check the consistency in the relationship between utterance duration and amount of linguistic content as measured by number of syllables. The underlying word forms were orthographically transcribed, with syllable boundaries marked by hyphens. In general, the transcription followed the spoken input as closely as possible to stay true to the child's exposure situation. Therefore, common dialectal variations were taken into account and transcribed as if they were words, and not in accordance with traditional Swedish orthography. Similarly, Swedish tense forms are sometimes colloquially shortened, as the last phoneme in present tense (*han ramla-r* = ‘he falls’) and the last unstressed syllable in past tense (*han ramla-de* = ‘he fell’) are dropped. These cases were consistently transcribed according to the audible number of syllables, although it generated one syllable less in the count. Unintelligible syllables were transcribed as ‘X’ and included in the syllable count. In total, the analyzed material contained 4618 turn-taking events.

## RESULTS

From the total number of turn-taking events, twenty-two containing the longest 0·5% of the pauses were defined as outliers and excluded from further analysis because of the skewed distribution of pause duration. The longest pause still included was 19·7 s. In total, 4596 turn-taking events were included in the analysis. The number of remaining turn-taking events in each category and the mean duration of the different turn-taking units are shown in Table 2 on group level. The following analyses are performed on group level, using all datapoints instead of mean values for each participant. Results for participant level are shown in Table 3.

**Table 2.** Mean (SD) of pause and utterance duration in different turn-taking events by vocabulary size group

Event type	Vocabulary size	No. of events	1st utterance duration (s)	2nd utterance duration (s)	Pause duration (s)
**Child–parent switching**	Large	657	0·74 (*0·51*)	1·09 (*0·69*)	0·51 (*1·07*)
	Typical	438	0·79 (*0·53*)	1·06 (*0·68*)	0·70 (*1·57*)
	Small	283	0·64 (*0·61*)	1·04 (*0·71*)	1·00 (*1·59*)
**Parent–parent intrapersonal**	Large	855	1·26 (*0·95*)	1·25 (*0·96*)	1·40 (*1·61*)
	Typical	956	1·25 (*0·84*)	1·27 (*0·87*)	1·63 (*1·82*)
	Small	1070	1·16 (*0·78*)	1·17 (*0·78*)	1·89 (*2·25*)
**Parent–parent switching**	Large	151	0·97 (*0·68*)	0·94 (*0·71*)	1·19 (*1·95*)
	Typical	59	1·10 (*0·72*)	1·01 (*0·71*)	0·88 (*1·19*)
	Small	127	1·07 (*0·72*)	0·95 (*0·68*)	1·87 (*2·50*)

**Table 3.** Mean (SD) of pause and utterance duration in different turn-taking events by vocabulary size group and participant

Event type	Vocabulary size	Participant	No. of events	1st utterance duration (s)	2nd utterance duration (s)	Pause duration (s)
**Child–parent switching**	Large	1	224	0·59 (*0·31*)	0·95 (*0·61*)	0·40 (*0·67*)
		2	126	0·86 (*0·52*)	1·19 (*0·65*)	0·48 (*0·72*)
		3	118	0·82 (*0·54*)	1·24 (*0·93*)	0·39 (*0·24*)
		4	81	1·02 (*0·85*)	1·22 (*0·67*)	0·89 (*2·35*)
		5	108	0·62 (*0·30*)	0·97 (*0·54*)	0·61 (*1·08*)
	Typical	6	131	0·78 (*0·34*)	1·09 (*0·56*)	0·40 (*0·47*)
		7	75	1·03 (*0·69*)	1·34 (*0·82*)	1·15 (*2·60*)
		8	76	0·81 (*0·66*)	1·02 (*0·74*)	1·24 (*2·45*)
		9	39	0·83 (*0·59*)	1·00 (*0·58*)	0·76 (*0·61*)
		10	117	0·64 (*0·42*)	0·91 (*0·64*)	0·38 (*0·47*)
	Small	11	28	0·81 (*0·87*)	1·03 (*0·67*)	1·51 (*2·61*)
		12	71	0·70 (*0·59*)	0·94 (*0·52*)	1·41 (*2·25*)
		13	75	0·38 (*0·24*)	1·03 (*0·80*)	0·91 (*0·91*)
		14	57	0·53 (*0·47*)	1·45 (*0·83*)	0·62 (*0·85*)
		15	52	0·98 (*0·78*)	0·76 (*0·44*)	0·71 (*0·84*)
**Parent–parent intrapersonal**	Large	1	125	1·15 (*0·75*)	1·21 (*0·67*)	1·58 (*2·01*)
		2	106	1·21 (*0·84*)	1·15 (*0·79*)	1·94 (*1·69*)
		3	205	1·25 (*0·99*)	1·27 (*0·91*)	1·19 (*1·29*)
		4	149	1·62 (*1·09*)	1·66 (*1·29*)	1·43 (*1·64*)
		5	270	1·15 (*0·92*)	1·07 (*0·89*)	1·24 (*1·51*)
	Typical	6	180	1·15 (*0·56*)	1·07 (*0·58*)	1·57 (*1·59*)
		7	173	1·25 (*0·73*)	1·22 (*0·69*)	1·75 (*1·54*)
		8	226	1·22 (*0·87*)	1·31 (*0·87*)	1·51 (*1·79*)
		9	227	1·40 (*1·03*)	1·44 (*1·09*)	1·96 (*2·49*)
		10	150	1·19 (*0·83*)	1·23 (*0·92*)	1·21 (*0·87*)
	Small	11	207	1·10 (*0·65*)	1·12 (*0·64*)	2·58 (*3·15*)
		12	164	0·84 (*0·52*)	0·79 (*0·43*)	2·41 (*2·71*)
		13	122	1·32 (*0·75*)	1·47 (*0·75*)	2·08 (*1·97*)
		14	355	1·36 (*0·92*)	1·34 (*0·91*)	1·23 (*1·19*)
		15	222	1·04 (*0·76*)	1·06 (*0·75*)	1·82 (*2·00*)
**Parent–parent switching**	Large	1	20	0·65 (*0·36*)	0·77 (*0·45*)	0·67 (*0·84*)
		2	2	1·62 (*0·71*)	1·11 (*0·06*)	2·37 (*2·02*)
		3	56	1·09 (*0·71*)	1·12 (*0·92*)	0·75 (*1·54*)
		4	25	1·23 (*0·90*)	0·93 (*0·56*)	1·12 (*1·84*)
		5	48	0·80 (*0·52*)	0·80 (*0·55*)	1·92 (*2·51*)
	Typical	6	5	0·81 (*0·39*)	0·71 (*0·31*)	0·61 (*0·56*)
		7	0	–	–	–
		8	54	1·13 (*0·74*)	1·04 (*0·73*)	0·91 (*1·23*)
		9	0	–	–	–
		10	0	–	–	–
	Small	11	49	1·08 (0·81)	0·98 (*0·74*)	1·38 (*1·60*)
		12	45	0·97 (*0·55*)	0·92 (*0·59*)	2·96 (*3·38*)
		13	0	–	–	–
		14	18	1·01 (*0·48*)	1·01 (*0·84*)	0·93 (*0·84*)
		15	15	1·40 (*1·03*)	0·89 (*0·58*)	1·36 (*2·14*)

### Temporal utterance duration and amount of linguistic content

In order to establish that there is no need to differentiate between temporal duration in utterances and measures of linguistic content, a linear regression predicting temporal utterance duration from number of syllables, turn-taking event type, and child vocabulary group (R^2^ = ·769; F_change_(4592,3) = 5084; *p* < ·001) was performed. Temporal duration of the second utterance was mostly predicted by number of syllables (final *β* = ·878; *p* < ·001), only marginally by turn-taking event type (final *β* = ·028; *p* < ·001), and not at all by child vocabulary group. Utterance duration therefore corresponds well to amount of linguistic content in utterances as measured by number of syllables.

### Utterance duration

Differences between mean utterance duration were tested using two-way ANOVAs, with child vocabulary size group (large, typical, or small) and turn-taking event type (child–parent switching, parent–parent intrapersonal, or parent–parent switching) as independent variables.

For first utterance duration, significant differences were found for different turn-taking event types (*F*(4587,2) = 183·64, *p* < ·001), and LSD post-hoc tests showed that all three types of events differed from each other (*p* < ·001). Shortest first utterance duration was found in child–parent switching events, whereas parent–parent intrapersonal events had the longest first utterance duration. No main effect was found for child vocabulary size group, nor was there any interaction between vocabulary size group and event type.

For second utterance duration, a significant difference was found for different turn-taking event types (*F*(4587,2) = 27·49, *p* < ·001), and the LSD post-hoc tests showed that all three types of event differed from each other (*p* < ·05). The longest second utterances were found in parent–parent intrapersonal events, whereas the shortest utterances were found in parent–parent switching events. There was no main effect of child vocabulary size group, and no interaction between event type and vocabulary size group.

### Pause duration

**Fig. 1.**
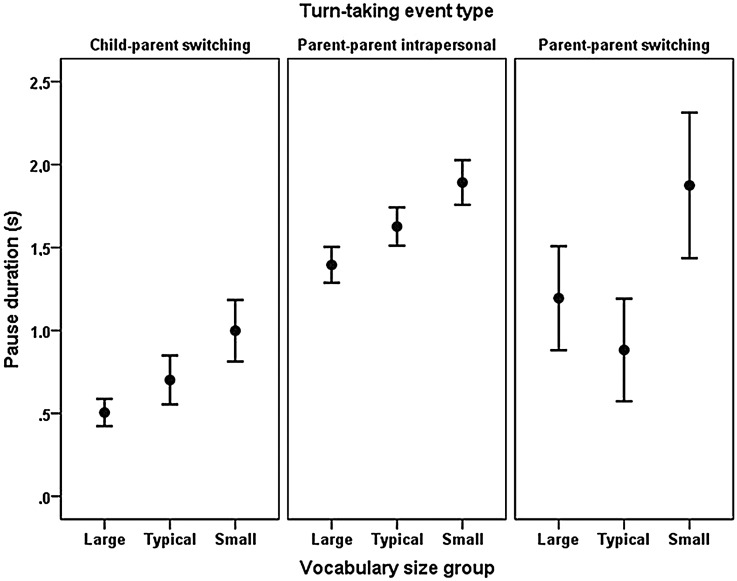
Mean (CI = 95%) pause duration in different turn-taking event types by vocabulary size group.

Differences between mean pause duration were tested using a two-way ANOVA, with child vocabulary size group (large, typical, or small) and turn-taking event type (child–parent switching, parent–parent intrapersonal, or parent–parent switching) as independent variables (see Figure 1).

Significant differences were found for different event types (*F*(4587,2) = 108·93, *p* < ·001), with LSD post-hoc tests revealing that all event types differed from each other. Both parent–parent event types contained longer pauses than the child–parent event type did (*p* < ·001). The duration of parent–parent switching pauses was also significantly longer than parent–parent intrapersonal pause duration (*p* = ·012). There was also a main effect of vocabulary size group (*F*(4587,2) = 22·25, *p* < ·001), and LSD post-hoc tests showed that all three groups differed in terms of pause duration (*p* < ·001). While no interaction was found between turn-taking event type and vocabulary size group, the longest pauses were found in the small vocabulary size group and the shortest pauses in the large vocabulary size group.

## DISCUSSION

First, we established that durational aspects of turn-taking events in speech are highly correlated to amount of linguistic content measured by number of syllables. Second, we found that the three vocabulary groups showed similar behaviour in terms of utterance duration. First utterances spoken by children were shorter than utterances spoken by adults. Adults typically produced longer utterances when responding to a child than when responding to another adult, but shorter utterances when responding to a child than when continuing a monologue. Finally, we showed that the mean pause duration differed between the three vocabulary size groups, with the longest duration in the small vocabulary size group and the shortest duration in the large vocabulary group. Furthermore, parents in all groups use shorter pauses in child–parent turn-taking events than they do in parent–parent turn-taking events.

Previous research has shown that the duration of utterances is typically shortened when adults speak to infants and children compared to when they speak to other adults (Fernald & Simon, 1984), or that there is no difference in utterance duration between IDS and ADS (Stern *et al.*, 1983). Our results contradict this, since parents in all three vocabulary groups used the shortest second utterances in a parent–parent switching event, that is, when they respond to an utterance of another adult. The most obvious explanation of this difference is that different speech characteristics are adjusted differently when speaking to children of different ages (Soderstrom, 2007). Fernald and Simon (1984) investigated IDS to neonates and Stern and colleagues explicitly tested the difference between ADS and IDS from when the infant was four months of age, whereas the children in our study were aged 1;6. Additionally, both the specific measures used as well as the setting of the interaction may account for further discrepancies between our and earlier results. Stern and colleagues (1983) looked at all of the mother's utterances (defined as periods of continuous vocalization separated by silences longer than 0·3 s), whereas we make a distinction between utterances (denoted as such by an experienced transcriber) depending on which kind of turn-taking event they are a part of. Even taking this into account and comparing second utterances from parent–parent intrapersonal events with those from parent–parent switching events as the closest approximation to the IDS versus ADS comparison of Stern *et al.* (1983) that we can manage, our results show parents using longer utterances when talking to children than when talking to adults. This may, however, be explained by the different settings for the interactions under investigation. Both studies cited above used speech recorded in laboratory settings, and separated the situations in which IDS/CDS and ADS were recorded. In our study, we specifically targeted the somewhat more disordered interactions of everyday situations recorded in the home of the family, and we separated utterances directed to children and adults from within the same interaction situation. Momentarily disregarding the age of the infant or child as a factor, the different results yielded in these settings give rise to the notion that durational aspects of ADS, specifically in relation to CDS or IDS, may differ between interactional settings; while mothers use longer utterances than they do to children when engaged in conversation with another adult, it seems that when the primary interactional focus is on the child, adult-directed utterances are in fact shorter than those directed to the child. This in turn gives rise to the interesting premise that the duration of utterances, regardless of to whom they are directed, may be highly dependent on who the current primary interactional partner of the speaker is. Studying this by combining the balanced situations of Stern and colleagues (1983) and the ecologically relevant naturalistic setting of our study would be of much interest.

When it comes to pauses, earlier studies have shown that pauses are generally longer in IDS and CDS than in ADS (Fernald & Simon, 1984), or that there is no difference between the two speech styles (Stern *et al.*, 1983). At first glance, our results seem to contradict those earlier results, since the shortest pauses in our data were found in parent–child switching events, and both types of parent–parent event have longer pauses. However, again taking the different interactional settings into account, we propose that the closest match to the IDS sample described in Stern *et al.* (1983) are the parent–parent intrapersonal turn-taking events in our dataset. It seems that occurrences like these – the same parent continuing speaking after a silence, potentially giving time for the child or infant to respond – fits the definition of pauses in the study by Stern and colleagues (1983). Thus, upon closer inspection, our results align with previous research on pauses in IDS in that for all vocabulary groups the parent–parent intrapersonal events contained longer pauses than did the switching parent–parent events. There is little previous data available on durational adjustments in speech to toddlers and children (the ages of the infants spoken to in the aforementioned studies are much younger than those in the present study), but our results suggest that pauses are longer in CDS than in ADS even when the child is as old as 1;6. In instances when the parent responds to a child vocalization however, the pauses are shorter than when they respond to an adult.

In terms of how parental temporal speech adjustments differ depending on the linguistic proficiency of the child, our results showed no difference between how parents with children in the different vocabulary size groups modified the durational aspects of their speech. Parents in all three vocabulary groups both used longer utterances when replying to children or continuing a monologue (assumed to be a part of the interaction with the child) than when speaking to adults, and left longer pauses when interacting with children and shorter pauses when replying to a child utterance. However, an overall difference between parents in the different vocabulary size groups was found in terms of pause duration: regardless of turn-taking event type, parents in the small vocabulary size group used longer pauses than parents in the other two groups, and parents in the typical vocabulary size group in turn used longer pauses than parents in the large vocabulary size group. Despite the fact that parents in all groups make similar modifications, the resulting effect is that the durational aspects of the language environment differ between vocabulary size groups: responses to children's vocalizations are not as rapid for children with smaller vocabularies than for those with larger vocabularies.

The present study is descriptive and as such no causal relationship can be inferred, but considering the influence of temporally contingent responses at earlier stages of language development (Goldstein *et al.*, 2003; Goldstein & Schwade, 2008), the present results give rise to interesting speculations on the relevance of temporal aspects of turn-taking for language development in toddlerhood. Rapid contingent parental responses to child communicative acts could well be beneficial for vocabulary development. These communicative acts are not limited to speech. Therefore, temporal contingency in communicative situations can be better analyzed using video-recordings of parent–child interactions, since that makes it possible to study parental responses to both verbal and non-verbal communicative acts from the child. To experimentally study the relevance of temporal contingency, word-learning computer games that simulate turn-taking situations with time-controlled responses could be used to determine whether response contingency impacts word learning.

## CONCLUSION

Communicative adjustments that are made by parents are similar across three vocabulary groups; that is, they use longer utterances and shorter pauses when responding to vocalizations from children at age 1;6 than when continuing a monologue or responding to another adult. However, the general speaking style also differs between groups in terms of pause duration. As such, the durational characteristics of turn-taking events that children are exposed to differ between vocabulary size groups, even though similar adjustments are made by parents in all groups. Parents of children with small vocabularies are not as rapid in their responses as parents of children with larger vocabularies. The possibility that responding rapidly to child vocalizations may be beneficial to language development is suggested as a worthwhile topic of future research.
